# The value of cuproptosis-related differential genes in guiding prognosis and immune status in patients with skin cutaneous melanoma

**DOI:** 10.3389/fphar.2023.1129544

**Published:** 2023-04-17

**Authors:** Yuming Sun, Shaorong Lei, Xiangyue Luo, Chufeng Jiang, Zhexuan Li

**Affiliations:** Department of Plastic and Cosmetic Surgery, Xiangya Hospital, Central South University, Changsha, Hunan, China

**Keywords:** cuproptosis, prognostic model, skin cutaneous melanoma, bioinformatics, tumor mutational burden (TMB)

## Abstract

**Background:** Skin cutaneous melanoma (SKCM) is one of the most common cutaneous malignancies, which incidence is increasing. Cuproptosis is a new type of programming cell death recently reported, which may affect the progression of SKCM.

**Method:** The mRNA expression data of melanoma were obtained from the Gene Expression Omnibus and the Cancer Genome Atlas databases. We constructed a prognostic model according to the cuproptosis-related differential genes in SKCM. Finally, real-time quantitative PCR was performed to verify the expression of cuproptosis-related differential genes in patients with different stages of cutaneous melanoma.

**Results:** We detected 767 cuproptosis-related differential genes based on 19 cuproptosis-related genes, and screened out 7 differential genes to construct a prognostic model, which including three high-risk differential genes (SNAI2, RAP1GAP, BCHE), and four low-risk differential genes (JSRP1, HAPLN3, HHEX, ERAP2). Kaplan-Meier analysis indicated that SKCM patients with low-risk differential genes signals had better prognosis. The Encyclopedia of Genomes results manifested that cuproptosis-related differential genes are not only involved in T cell receptor signaling channel, natural killer cell mediated cytotoxicity, but also chemokine signaling pathway and B cell receptor signaling pathway. In our risk scoring model, the receiver operating characteristic (ROC) values of the three-time nodes are 0.669 (1-year), 0.669 (3-year) and 0.685 (5-year), respectively. Moreover, the tumor burden mutational and immunology function, cell stemness characteristics and drug sensitivity have significant differences between low-risk group and high-risk group. The mRNA level of SNAI2, RAP1GAP and BCHE in stage Ⅲ+Ⅳ SKCM patients was significantly higher than that in stage Ⅰ+Ⅱ patients, while the level of JSRP1, HAPLN3, HHEX and ERAP2 in stage Ⅰ+Ⅱ SKCM patients was more remarkable higher than that in stage Ⅲ+Ⅳ SKCM patients.

**Conclusion:** In summary, we suggest that cuproptosis can not only regulate the tumor immune microenvironment but also affect the prognosis of SKCM patients, and may offer a basic theory for SKCM patients survival studies and clinical decision-making with potentially therapeutic drugs.

## Introduction

Skin cutaneous melanoma (SKCM) is the most frequent, malignant and aggressive cutaneous malignancies, which is the result of genetic alterations caused by complex interactions between genetic, environmental and other factors (Andrzej et al., 2001; Xiong et al., 2022). The global incidence of SKCM is increasing rapidly. According to the International Agency for Research on Cancer (IARC), the number of new cases of SKCM worldwide increased from 287,000 to 324,000 between 2018 and 2020 (Bray et al., 2018; Sung et al., 2021). Although the discovery of new targeted drugs and therapeutic targets has improved the treatment effect of SKCM, the annual number of new deaths due to SKCM has decreased, but the number of new deaths of more than 57,000 is still unacceptable (Lorentzen, 2019; Jenkins and Fisher, 2021; Sung et al., 2021). Melanoma cells pose a therapeutic challenge by exploiting their intrinsic resistance to apoptosis against a variety of chemotherapy agents (Hussein et al., 2003; Soengas and Lowe, 2003). The emergence of biological sequencing analysis technology has found that many biomarkers may be related to melanoma (Gogas et al., 2009). The discovery of new biomarkers and the elucidation of their relationship with melanoma are crucial for the early diagnosis and prognosis evaluation of SKCM.

An appropriate amount of copper is an essential element for human survival, and it is mainly involved in pathways required for normal cell development and metabolism (Huang et al., 2022). The function of important metal binding enzymes will be impaired if copper is too little, while excessive amounts can lead to cell death (Martha and Scott, 2022). Recently, Tsvetkov et al. (2022) reported a new mode of cell death that caused by copper, which distinct from apoptosis, pyroptosis, and ferroptosis. Excess copper leads to aggregation of fatty acylated proteins via direct bond to lapidated ingredients in the tricarboxylic acid (TCA) cycle, resulting in loss of iron-sulfur clusterin, proteotoxic stress and eventually cell death (Tsvetkov et al., 2022). The novel mechanism of cuproptosis proposed by Tsvetkov may provide a new way to exploit the unique effects on this metal to kill cancer cells, especially in cancers that are naturally resistant to apoptosis (Martha and Scott, 2022).

We hypothesized that cuproptosis is closely related to the treatment and prognosis of melanoma. In our research, we first constructed a prognostic model based on the Cancer Genome Atlas (TCGA) database and the Gene Expression Omnibus (GEO) database to analyze the cuproptosis-related differential gene data in SKCM.

## Materials and methods

### Data collection and preparation

The GSE65904 from the Gene Expression Omnibus (https://www.ncbi.nlm.nih.gov/geo/) database which include 214 specimens of SKCM patients were downloaded. Another RNA sequence transcriptome analysis dataset includes 469 melanoma samples that were obtained from TCGA (https://portal.gdc.cancer.gov/projects/TCGA-SKCM). The age, sex, grade, survival time, survival status, tumor stage and other clinical data of SKCM patients were also obtained from TCGA. Additionally, we acquired 19 genes related to cuproptosis from previous studies, including CDKN2A, NLRP3, ATP7A, ATP7B, MTF1, SLC31A1, GLS, PDHA1, DLD, NFE2L2, DBT, DLAT, PDHB, DLST, LIPT1, LIPT2, LIAS and GCSH (Tsvetkov et al., 2022).

### Consensus clustering analysis of cuproptosis-related genes to screen the cuproptosis-related differential genes

Based on the SKCM RNA sequence transcriptome in TCGA and GEO, We used the “ConsensusClusterPlus” package to classify SKCM patients into different clusters base on the consensus expression of cuproptosis-related genes (Wilkerson and Hayes, 2010). The cluster variable (k) was increased from 2 to 9 to find the most appropriate K value, and 683 SKCM patients were divided into appropriate clusters according to the expression similarity of 17 cuproptosis-related genes. Kaplan-Meier (KM) survival analysis was applied to parabole the survival times of patients divided into two clusters of melanomas which the “survival” package. The differential genes related to cuproptosis were identified by comparative analysis of the two clusters. The age, sex, and expression of copper death-related genes in 683 SKCM patients were presented using the “pheatmap” package to visualize differences between clinical parameters and taxonomic clusters.

### Pathway enrichment analysis and gene set enrichment analysis

In order to clarify the biological function of cuproptosis-related differential genes in SKCM, Gene ontology (GO) is used to analyze biological functions, while enrichment pathways are achieved through the Encyclopedia of Genomes (KEGG).

### Cuproptosis-related differential genes prognostic model construction for SKCM

Consensus clustering analysis of cuproptosis-related differential genes. Then, to construct a cuproptosis-related differential genes risk prognostic model, we performed the least absolute shrinkage and selection operator (LASSO) regression analyses for cuproptosis-related differential genes associated with differential prognosis in SKCM patients. We use the R package “glmnet” to establish (LASSO) Cox regression model, which can screen out potential cuproptosis-related differential genes and create a prognosis model for SKCM patients (Engebretsen and Bohlin, 2019).

### Validation of risk models

We divided melanoma patients into two groups according to the cuproptosis score in the model: low-risk group and high-risk group. After comparing the difference of overall survival rate between the two groups, the specificity and sensitivity of the risk model were evaluated by the receiver operating characteristic (ROC) curve. Draw ROC curves of each group for 1-year, 3-year and 5-year, respectively.

### Independent predictive analysis of the risk models

Univariate and multivariate Cox regression analysis was performed combining the clinical characteristics of melanoma patients and the risk assessment model of cuproptosis-related differential genes. The result of multivariate Cox analysis *p* < 0.05 would be considered as a special prognostic factor for melanoma prognosis.

### Analysis of tumor burden mutational and immunological function based on cuproptosis-related differential genes risk score

The nucleotide mutation data related to melanoma patients were collected from TCGA and GEO database. We used the “limma, pheatmap, ggpubr” packages to construct heatmaps and violin plot, which show the differences level of immune cell infiltration and tumor immune microenvironment. Next, in order to predict the tumor purity in TME, ESTIMATE was used to estimate the stromal cells and immune cells in melanoma tissue (Hou et al., 2022). Get the scores of StromalScore, ImmuneScore and ESTIMATEScore of the melanoma tissue, and analyze different groups (high-risk/low-risk groups)with “Limma” packages. Then, utilize the “maftools” package to divide the tumor mutational burden (TMB) in melanoma samples into low-risk groups and high-risk groups base on the predicted model risk score.

### Evaluation of drug sensitivity

IC50 represents the half inhibitory concentration of the tested antagonist. To assess the potential clinical utility of cuproptosis-related differential genes in SKCM treatment. We tested each melanoma patient’s response to sensitivity to several chemotherapy drugs using the Cancer Drug sensitivity Genomics (GDSC; https://www.cancerrxgene.org/) database and quantified IC50 with the R’s “pRRophetic” package. We performed Wilcoxon symbolic rank test to compare IC50 differences between low-risk and high-risk groups of commonly used antitumor agents. Boxplots were drawn using the R package “ggplot2.”

### Clinical specimens and real-time quantitative PCR

Tumor tissue was obtained from patients diagnosed with SKCM who underwent surgery in our hospital between January 2021 and December 2022. All patients signed informed consent prior to use of clinical specimens. The tumor tissues used in this study were approved by the Ethics Committee of Xiangya Hospital, Central South University. Total tissue RNA was extracted with Trizol (Thermo Fisher Scientific) base on the standard protocol. Next, total RNAs were used to synthesize cDNA with HiScript Q RT SuperMix kit (Vazyme, Nanjing, China). Subsequently, Quantitative PCR analyses were conducted with SYBR Green qPCR Master Mix (Bimake). GAPDH was used as an endogenous control. The primer sequences are listed in Table 1.

**TABLE 1:** The primers for 7 cuproptosis-related differential genes.

JSRP1	Forward: 5′ TGT​CGC​TCA​ACA​AGT​GCC​TG
	Reverse: 5′
	GCC​TGG​GCC​TCG​AAC​TTA​G
HAPLN3	Forward: 5′
	TGC​GTG​TCA​AAT​GGT​GGA​AG
	Reverse: 5′
	GTA​ACG​CCC​ATA​GTC​CTC​CAG
SNAI2	Forward: 5′
	CGA​ACT​GGA​CAC​ACA​TAC​AGT​G
	Reverse: 5′
	CTG​AGG​ATC​TCT​GGT​TGT​GGT
HHEX	Forward: 5′
	ACG​CCC​TTT​TAC​ATC​GAG​GAC
	Reverse: 5′
	CGT​GTA​GTC​GTT​CAC​CGT​C
RAP1GAP	Forward: 5′
	GCC​CAC​AAC​CAA​GGT​GAA​G
	Reverse: 5′
	CTG​GAC​AAC​ATT​AGG​GAA​CTC​G
ERAP2	Forward: 5′
	CCA​GAG​AAA​CTT​ACG​CCT​CAC
	Reverse: 5′
	GCC​TGG​GTT​GGC​TCA​AAA​TC
BCHE	Forward: 5′
	GTC​AGA​GGG​ATG​AAC​TTG​ACA​G
	Reverse: 5′
	TGA​ATC​GAA​GTC​TAC​CAA​GAG​GT

### Statistical analysis

All statistical analyses were performed in R software (version 4.2.1), and values of *p* < 0.05 were considered statistically significant.

## Result

### The genetic characteristics and transcription changes of cuproptosis-related genes in SKCM

**FIGURE 1:**
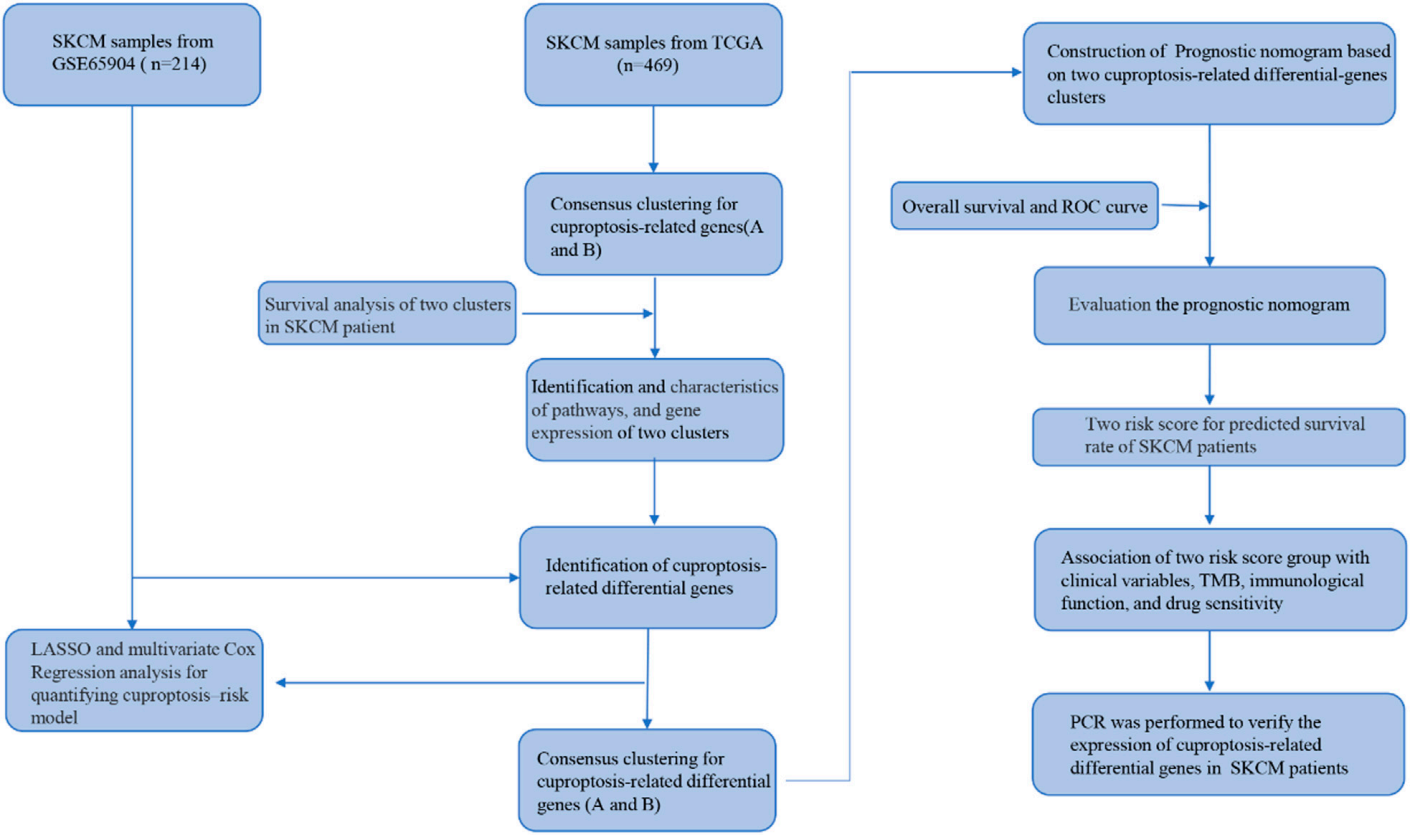
A flow chart showing the analysis details.

**FIGURE 2:**
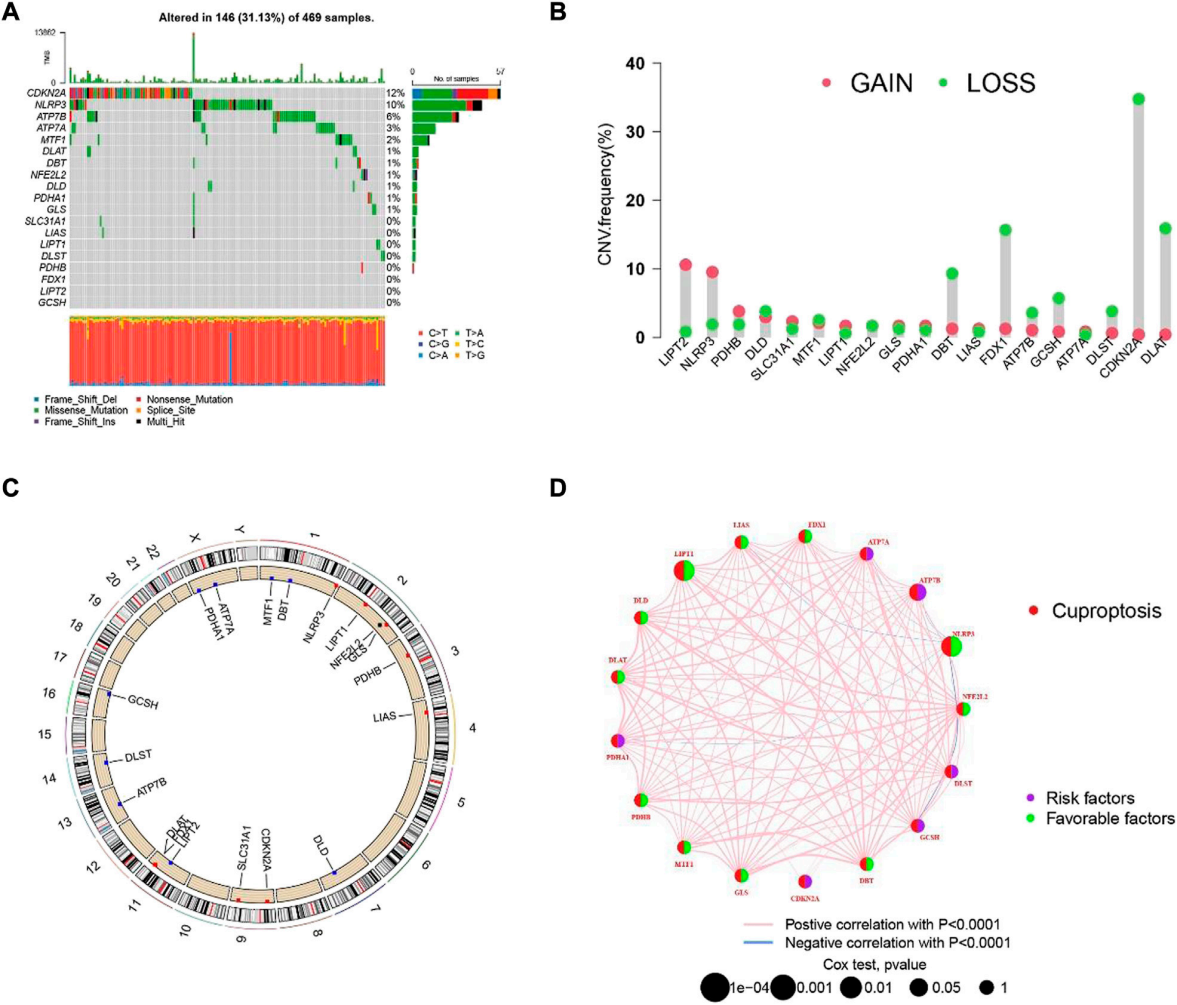
The hereditary characteristics and transcription changes of cuproptosis-related genes in melanoma. **(A)** Genetic alteration of cuproptosis-related genes in melanoma. **(B)** Cuproptosis of CNV gain, loss, and non-CNV among cuproptosis-related genes. **(C)** The circus plots show the position of cuproptosis-related genes in chromosomes. **(D)** The PPI network shows the prognosis of cuproptosis-related genes.

The process of our research is shown in Figure 1. Firstly, based on TCGA database, a total of nineteen cuproptosis-related genes were identified in SKCM transcription group data. The waterfall map showed a mutation of 146 (31.13%) of the 469 SKCM samples. At the same time, the CDKN2A mutation rate was the highest among nineteen cuproptosis-related genes (Figure 2A). Next, we examined the copy number variations (CNV) in these nineteen cuproptosis-related genes and found that the CNV of LIPT2 and NLRP3 increased significantly, while extensive decrease in CNV for DBT, FDX1, CDKN2A, and DLA (Figure 2B). As shown in Figures 2C, D, the position of cuproptosis-related genes in chromosomes. In the survival analysis of the 19 cuproptosis-related genes in melanoma patients, seventeen cuproptosis-related genes have significant differences, while LIPT1 and NLRP3 showed the most significant differences (Supplementary Figure S1). Moreover, we found that LIPT1 and NLRP3 have the strongest correlation with SKCM patient’s prognosis through protein-protein interaction (PPI) analysis.

### Cuproptosis-related differential genes are identified by consensus clustering

**FIGURE 3:**
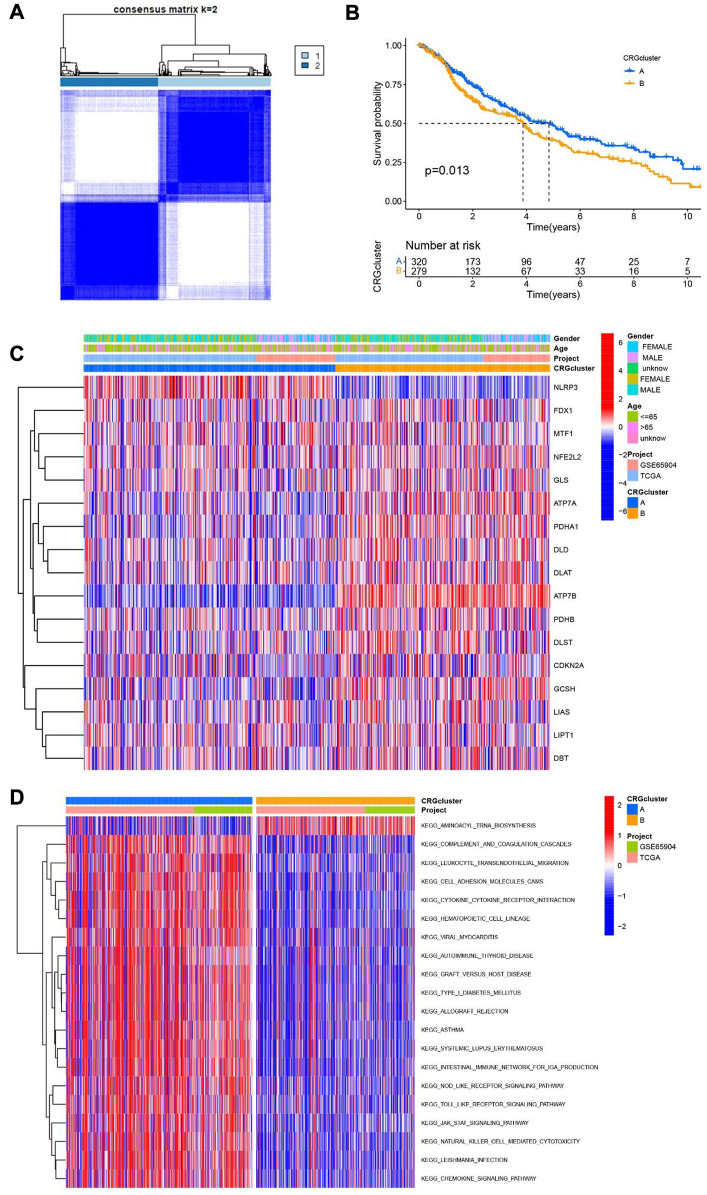
Two clusters of cuproptosis-related genes. **(A)** The Consensus matrix heatmap defined two clusters based on the consistency of cuproptosis-related genes expression (K = 2). **(B)** Compare the survival time of two clusters through KM survival analysis. **(C)** Different expression of the two clusters of cuproptosis-related genes clusters. **(D)** KEGG analysis of two clusters of cuproptosis-related genes.

In order to understand the role of cuproptosis in melanoma, we obtained 469 and 214 melanoma patients from TCGA and GEO databases respectively. Then according to the consistency of mRNA expression of cuproptosis-related genes and molecular clustering effects to found the optimal k. We found the k = 2 is the best way to cluster in k = 2 to 9 in OS, and there were significant differences in survival analysis between the two clusters (*p* = 0.013) (Figures 3A, B). The cuproptosis-related genes between the two clusters showed significant differences in the heatmap (Figure 3C). To assess the signaling pathways associated with cuproptosis-related genes, the functional enrichment of significantly different between the two clusters in KEGG was conducted (Figure 3D). Furthermore, there were significant differences in the relationship between cuproptosis-related genes and immune cells in the two clusters (Supplementary Figure S2).

### Enrichment analysis of cuproptosis-related genes in melanoma

**FIGURE 4:**
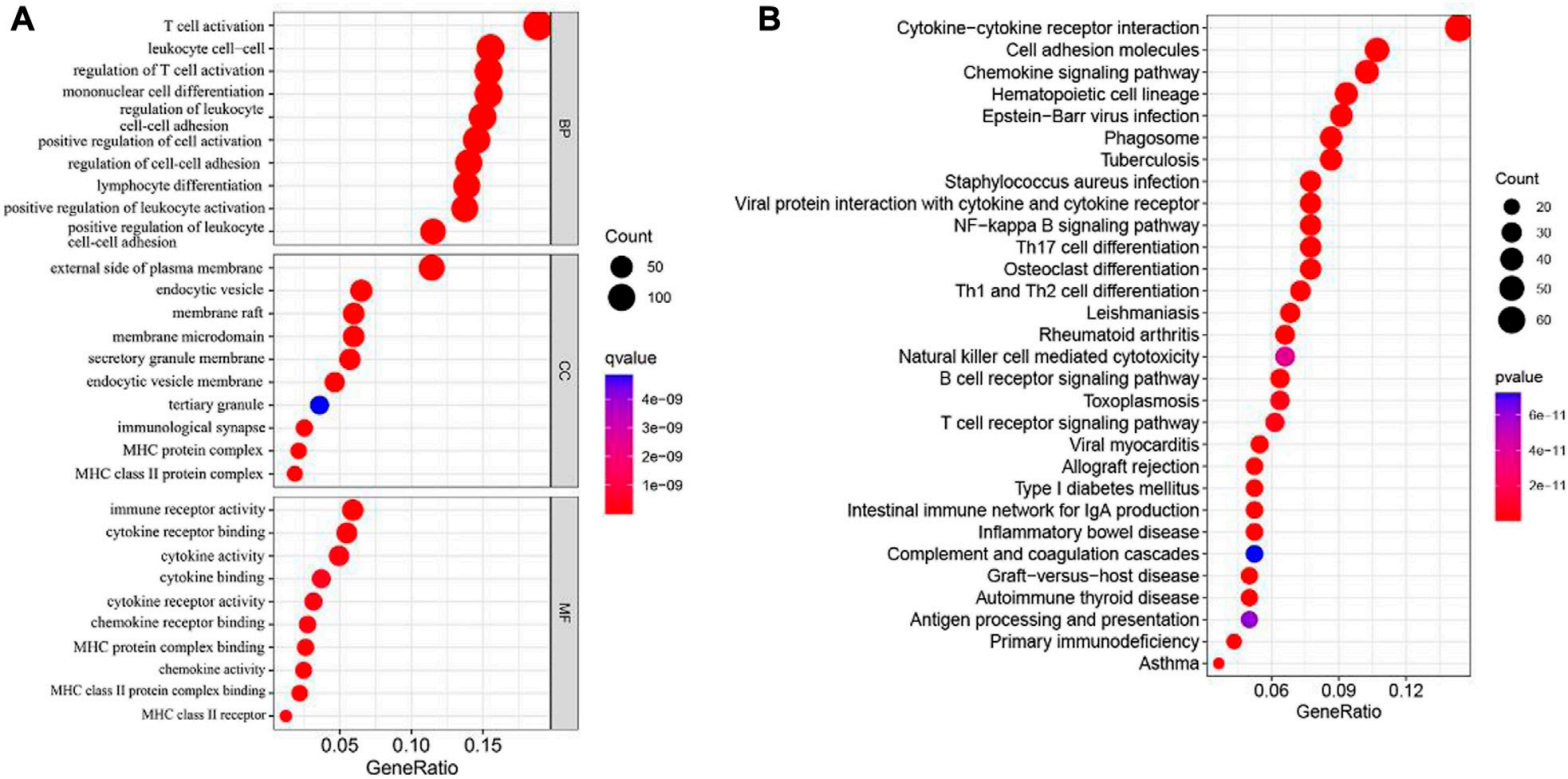
GO and KEGG analyses for cuproptosis-related differential genes in melanoma. **(A)** GO analysis. **(B)** KEGG analysis.

To further understand the downstream reasons, a total of 767 genes was correlated with cuproptosis were identified. Then, we conducted GO and KEGG enrichment analysis for purpose of knowing the potential functions of cuproptosis-related differential genes in SKCM patients. GO analysis shows that the biological process category (BP), the cuproptosis-related differential genes are mainly concentrated in T cell activity, positive regulation of cell, mononuclear cell and leukocyte cell−cell adhesion. From the cell component (CC), we found that these genes were basically involved in the external side of plasma membrane, endocytic vesicle, membrane raft and membrane microdomain. Moreover, In the molecular function (MF) category, they mostly participate in immune regulation and cytokine functions, for instance immune receptor activity and cytokine binding (Figure 4A). In addition, the KEGG results manifested that cuproptosis-related differential genes are mainly involved in T cell receptor signaling pathway, Cytokine−cytokine receptor interaction, chemokine signaling pathway and T cell receptor signaling pathway (Figure 4B).

### Construction of a cuproptosis-related differential genes risk prognostic model

**FIGURE 5:**
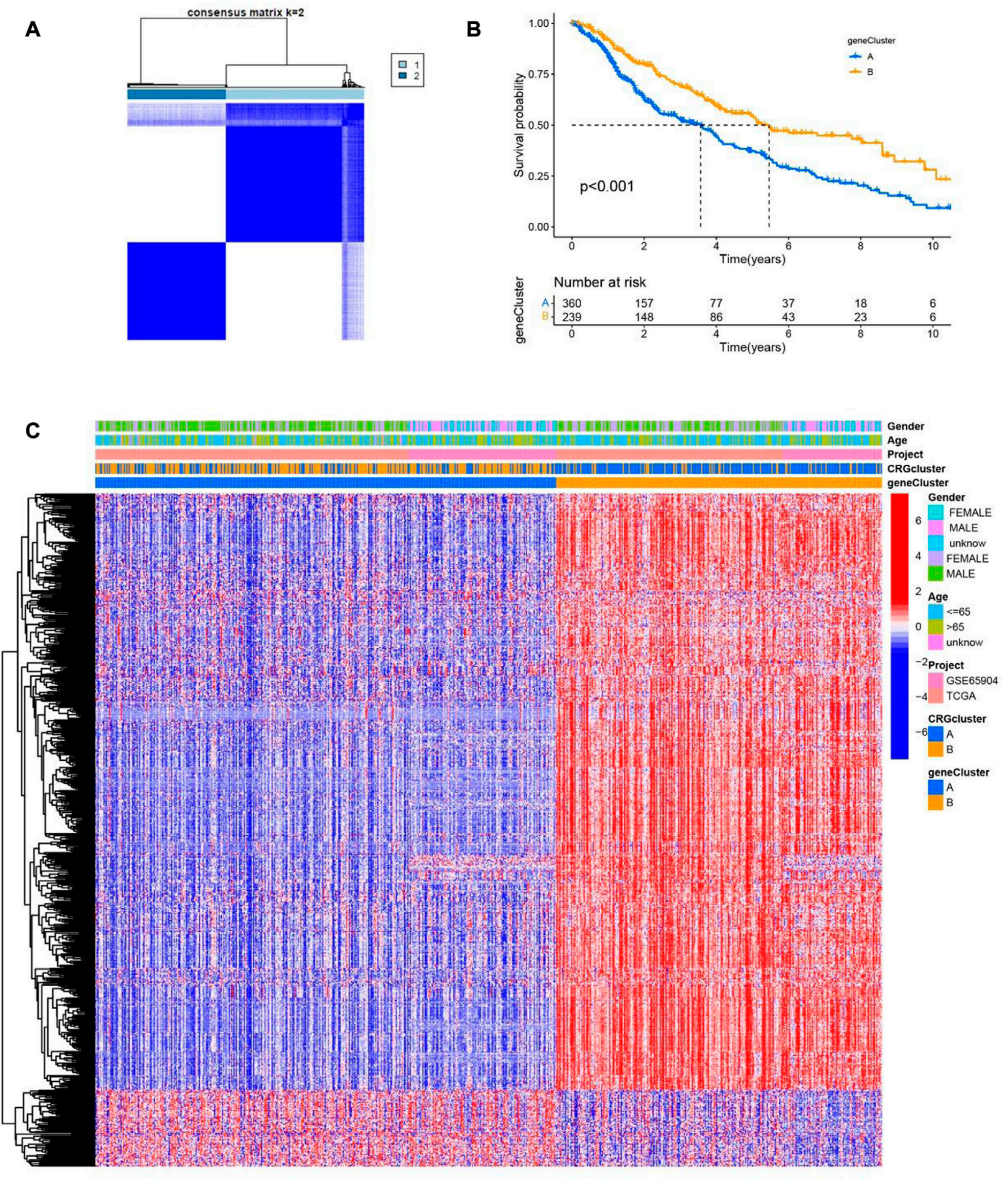
Two clusters of cuproptosis-related differential genes. **(A)** The Consensus matrix heatmap defined two clusters based on the consistency of cuproptosis-related differential genes expression (K = 2). **(B)** Compare the survival time of two clusters through KM survival analysis. **(C)** The clinical and pathological information of two cuproptosis-related differential genes clusters are shown in the heatmap.

According to the consistency of mRNA expression of cuproptosis-related differential genes, Consensus Cluster Plus package was used to consensus clustering analysis. The best stability cluster was K = 2 which was chosen to consensus cluster (Figure 5A). The survival rate between the two clusters showed significant difference in Kaplan Meier curve (*p <* 0.001) (Figure 5B). The heatmap shows that the clinical pathological characteristics between the two clusters are significantly different. Compared with cluster B, most of cuproptosis-related differential genes were higher in cluster A (Figure 5C).

**FIGURE 6:**
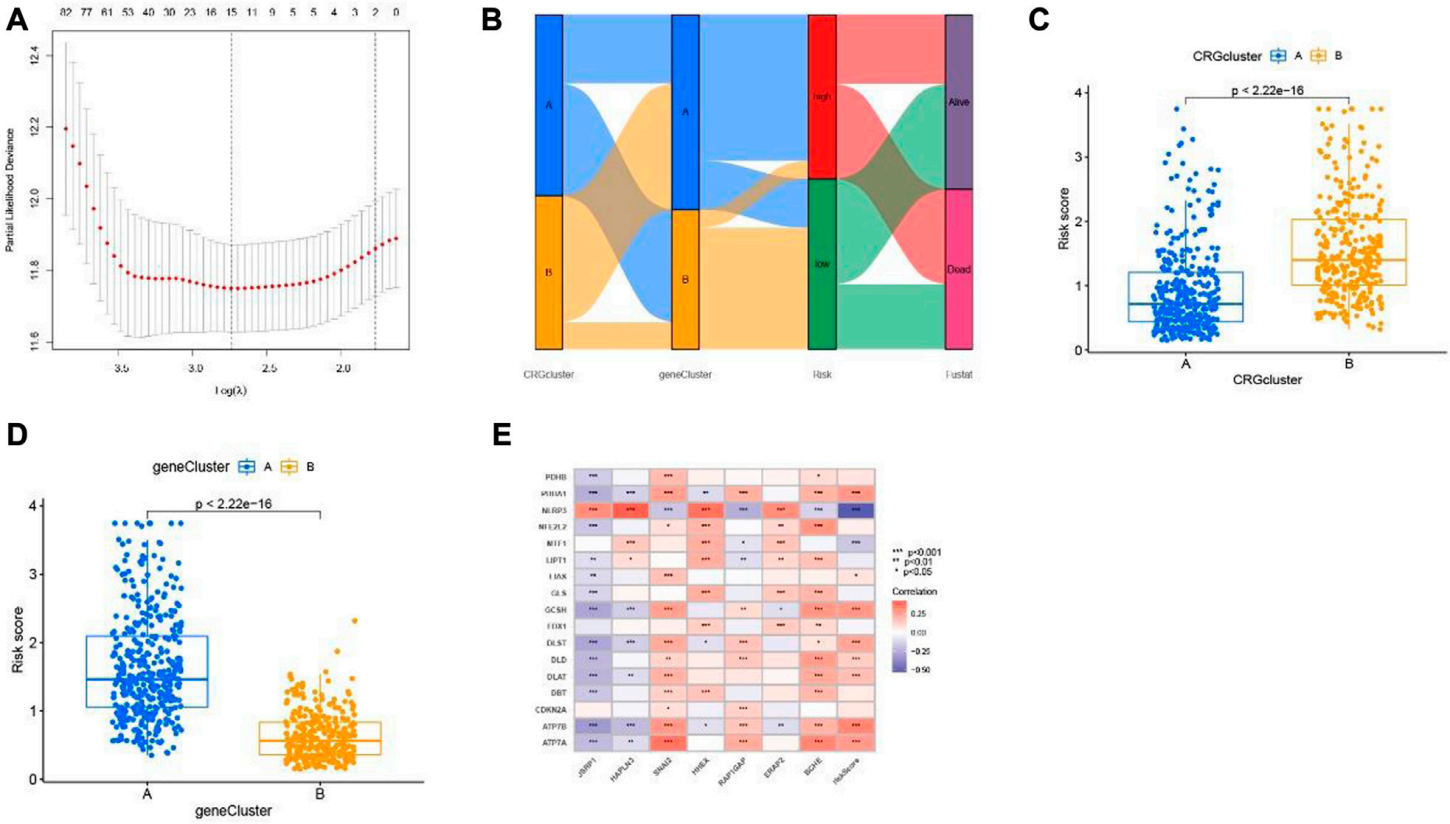
Cuproptosis-related differential genes risk prognostic model. **(A)** LASSO Cox regression analysis showed cuproptosis-related differential genes. **(B)** Sankey plot of two risk group anlysis. **(C)** two cuproptosis subtypes and **(D)** two gene clusters. **(E)** Association between cuproptosis-related genes and seven cuproptosis-related differential genes constructed the nomogram.

Then, the LASSO algorithm builds a prognostic model based on melanoma patients based on 7 cuproptosis-related differential genes (Figure 6A, Supplementary Figure S3). The Sankey plot shows the distribution of the cuproptosis subgroups, two gene clusters, cuproptosis score, and the survival status of melanoma patients (Figure 6B). Moreover, we also found that the scores of cuproptosis clusters and gene clusters were significantly different (*p <* 0.001) (Figures 6C, D). Finally, the correlation between seven cuproptosis-related differential genes and 17 cuproptosis-related genes was shown in the nomogram (Figure 6E).

### Prognostic evaluation of cuproptosis-related differential genes nomogram in SKCM patients

**FIGURE 7:**
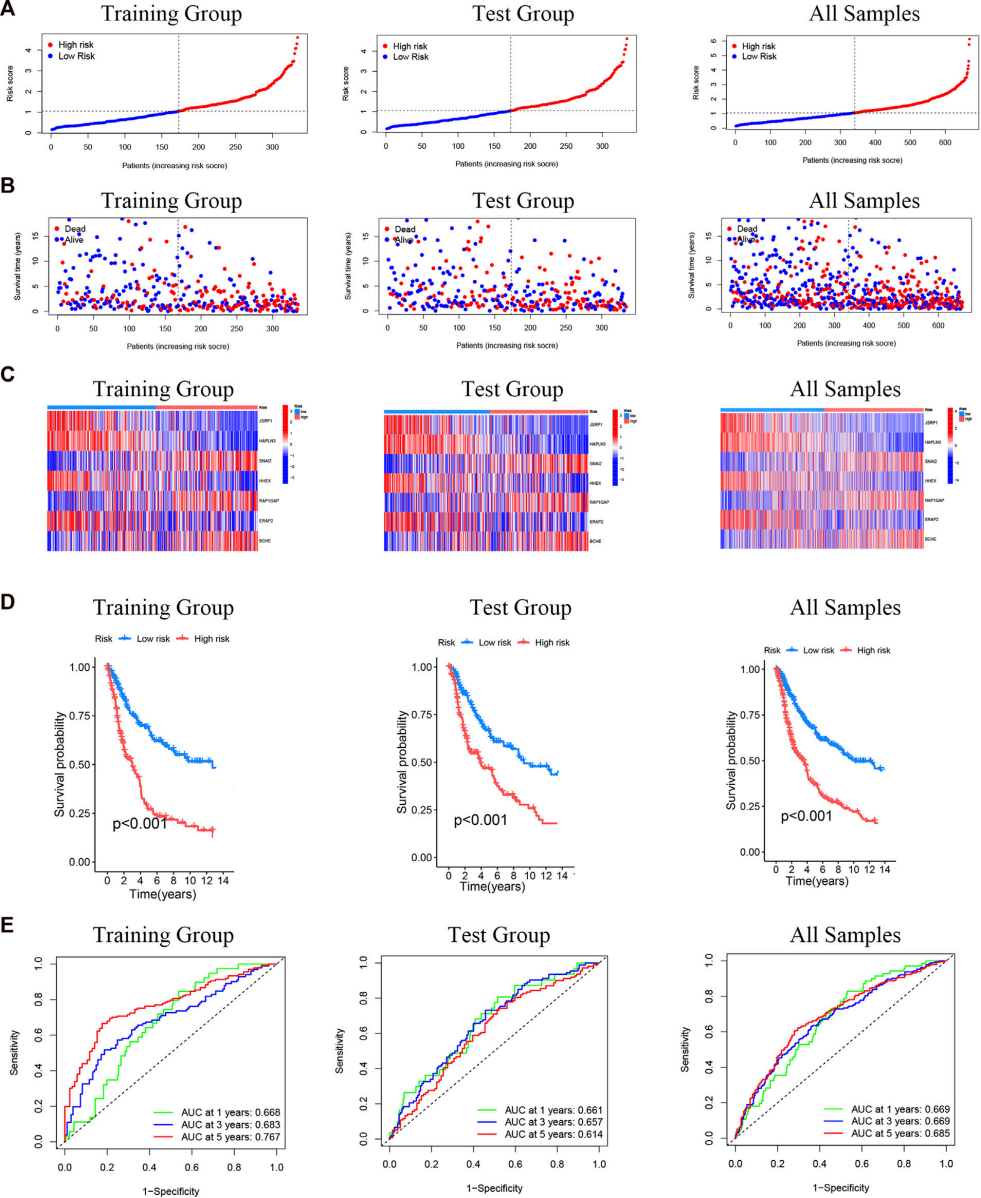
Prognostic evaluation of cuproptosis-related differential genes nomogram in melanoma patients. **(A)** Risk score distribution of two risk groups. **(B)** The scatter chart shows the distribution of OS and different risk groups. **(C)** The expression of the seven cuproptosis-related differential genes in the risk model. **(D)** K-M survival curve of melanoma patients in different risk scoring groups. **(E)** ROC curves for training group and test group (including all samples).

We calculated a risk score for every SKCM patient sample according to the previously obtained model of seven differential genes associated with cuproptosis. Patients are then classified as low risk (risk score below the median risk score) and high risk (risk score above the median risk score) (Figure 7A). It can be observed from the scatter plot of the risk score distribution that with the increase of the risk score, the mortality of the high-risk group also increases, while fewer deaths occur in the low-risk group (Figure 7B). The expression of 7 cuproptosis-related differential genes signals in SKCM was consistent among training group, test group and the all-samples group (Figure 7C). To assess the prognostic prediction of our model for OS status in SKCM patients, we performed K-M survival curve plotting, which showed that the high-risk group had significantly lower OS than the low-risk group in both the training and testing groups (including all samples) (*p* < 0.001; Figure 7D). The ROC curve analysis was performed to assess the accuracy of the prediction model, and the AUC values of the ROC curves of all samples were 0.669(1 year), 0.669(3 years), and 0.685 (5 years), respectively, which were consistent with the results of training group and test group (Figure 7E). These results suggest that these seven genes are associated with cuproptosis and that this model can predict prognosis in SKCM patients.

### Evaluation of the clinical utility of cuproptosis-related differential genes nomogram

**FIGURE 8:**
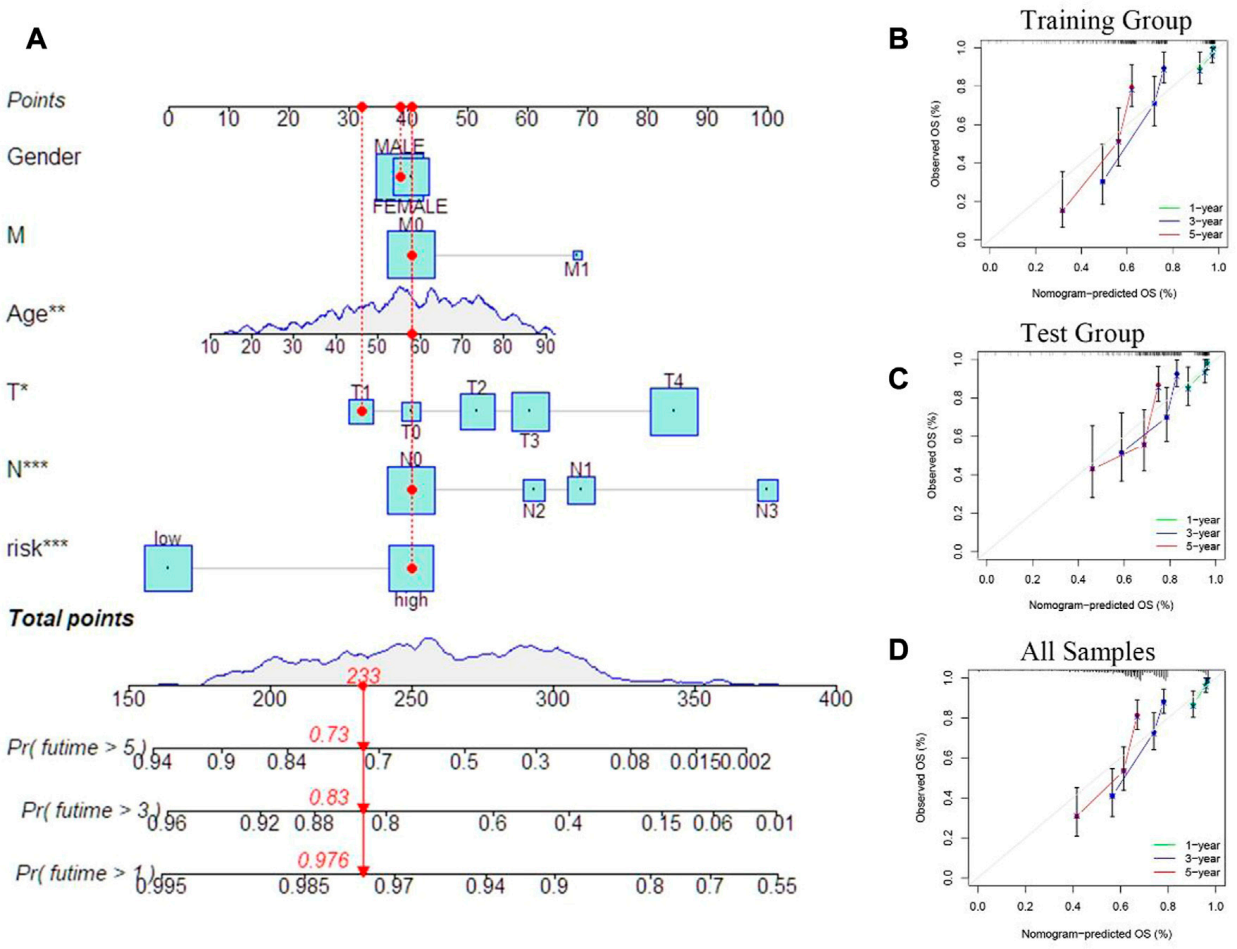
Prognostic model evaluation. **(A)** Nomogram with risk score and clinical characteristics of melanoma patients. **(B–D)** Calibration curves show the difference between the 1 -, 3 -, and 5-year operating systems predicted by the training and test groups (including all samples) and the actual operating systems.

In order to predict the clinical outcomes in patients of SKCM, a nomogram plot was used to perform quantitavive analysis. As shown in Figure 8A, the calculated risk score increased, the predicted survival rate of SKCM patients decreased. Cuproptosis-related differential genes prognosis model, which is to exert a positive effect in the clinical diagnosis and treatment in SKCM, based on this, to assess the clinical potential applications of this model, we developed a risk score and SKCM patients’ clinical characteristics of nomograph, for which to predict the SKCM patients’ 1 year, 3 years and 5 years OS rates. Furthermore, calibration curves were constructed to assess whether he actual observed OS rates were consistent with the nomogram-predicted OS rates. The results showed that 1-, 3-, and 5-year OS predictions between the actual observation group (all samples), training group, and test group were relatively good (Figures 8B–D).

### Tumor burden mutational study and immunological function based on cuproptosis-related differential genes in SKCM samples

**FIGURE 9:**
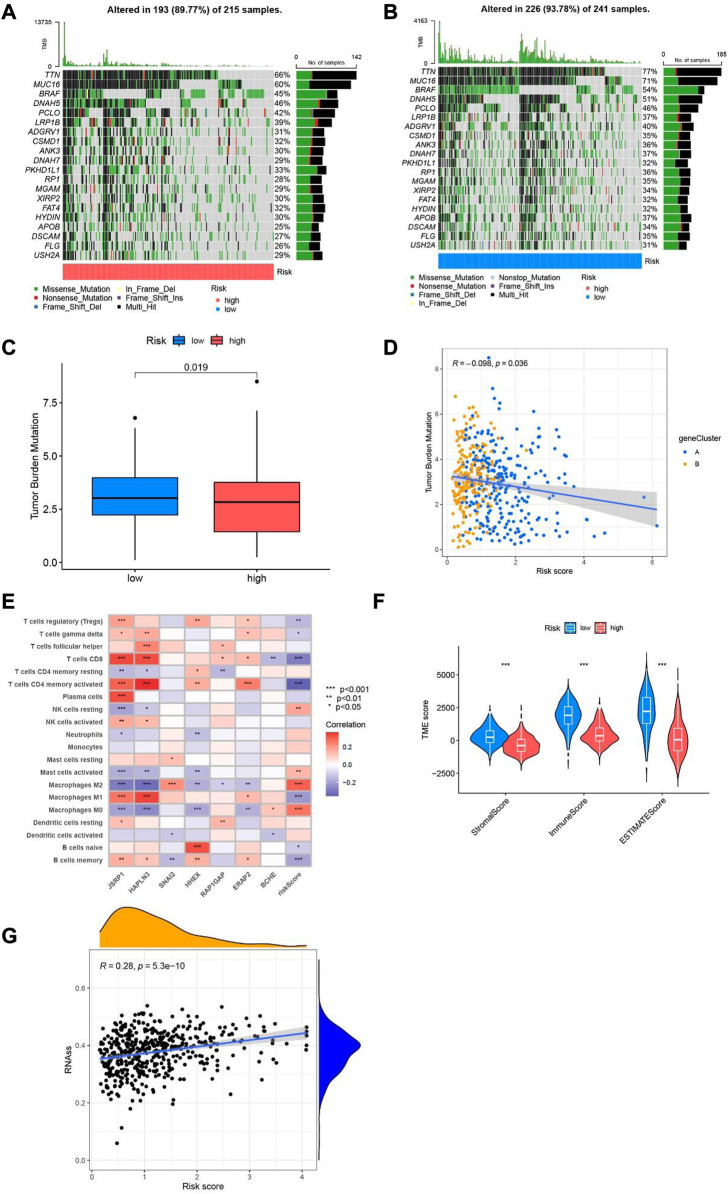
Tumor mutation load and immune function in different risk population. **(A)** Waterfall plot of mutant genes in the high-risk group. **(B)** Waterfall plot of mutant genes in the low-risk group. **(C)** boxplot depict the tumor mutational burden of melanoma patients in the two groups. **(D)** The correlation analysis of risk score and tumor burden mutational in melanoma. **(E)** Heatmap of the association of 7 cuproptosis-related differential genes with immune cells. **(F)** Violin diagram of StromalScore, ImmuneScore and ESTIMATEScore compared between low-risk group and high-risk group. **(G)** Relevance analysis between risk score and stemness characteristics.

Primarily, according to the transcriptional information of melanoma obtained from TCGA and GEO, calculated the tumor mutation burden index of genes low-risk group and in high-risk group respectively. The with the top 20 genes with highest mutation frequency in the low and high-risk group are described in the waterfall diagram. In the high-risk group (Figure 9A), 193 of the 215 samples found mutations; in the other group (Figure 9B), 226 mutations occurred in 241 samples. Among the mutated genes, MUC16, DNAH5, PCLO, TTN, LRP1B, BRAF, ADGRV1, CSMD1, ANK3, DNAH7, PKHD1L1, RP1, MGAM, XIRP2, FAT4, HYDIN, APOB, DSCAM, FLG and USH2A occurred simultaneously between the low- and high-risk groups. TTN, MUC16 and BRAF had the highest mutation frequencies in the two groups, and the mutation frequencies were77%, 71% and 54% in the low-risk group and 66%, 60% and 45% in the high-risk group, respectively. The boxplot showed that the mutation of tumor load was significantly lower in patients with high scores than in patients with low scores (*p* = 0.019, Figure 9C). We use the Pearson related analysis to confirm the correlation between Risk Score and Tumor Burden Mutational in melanoma patients, and verify the negative correlation between risk score and Tumor Burden Mutational (*p* = 0.036, R = −0.098, Figure 9D). Next, we explored the correlation between the seven cuproptosis-related differential genes included in the model and immune cells, it was observed that the expression of seven cuproptosis-related differential genes was strongly related to CD8+T cells, T cells CD4 memory activated, Macrophages M0-2. Especially in the expressions of JSRP1 and HAPLN3 are more closely related to immune function (Figure 9E). Then, the Matrix scores, immune scores, and ESTIMATE scores in SKCM samples were evaluated through ESTIMATE methods, and the scores between different groups were compared. We found that the high -risk group was lower than the low -risk group, with significant differences (Figure 9F). Furthermore, we analyzed the relationship between risk score and stemness characteristics in SKCM, and determined that risk score is positively correlated with stemness characteristics (*p <* 0.001, R = 0.28, Figure 9G).

### Drug sensitivity

**FIGURE 10:**
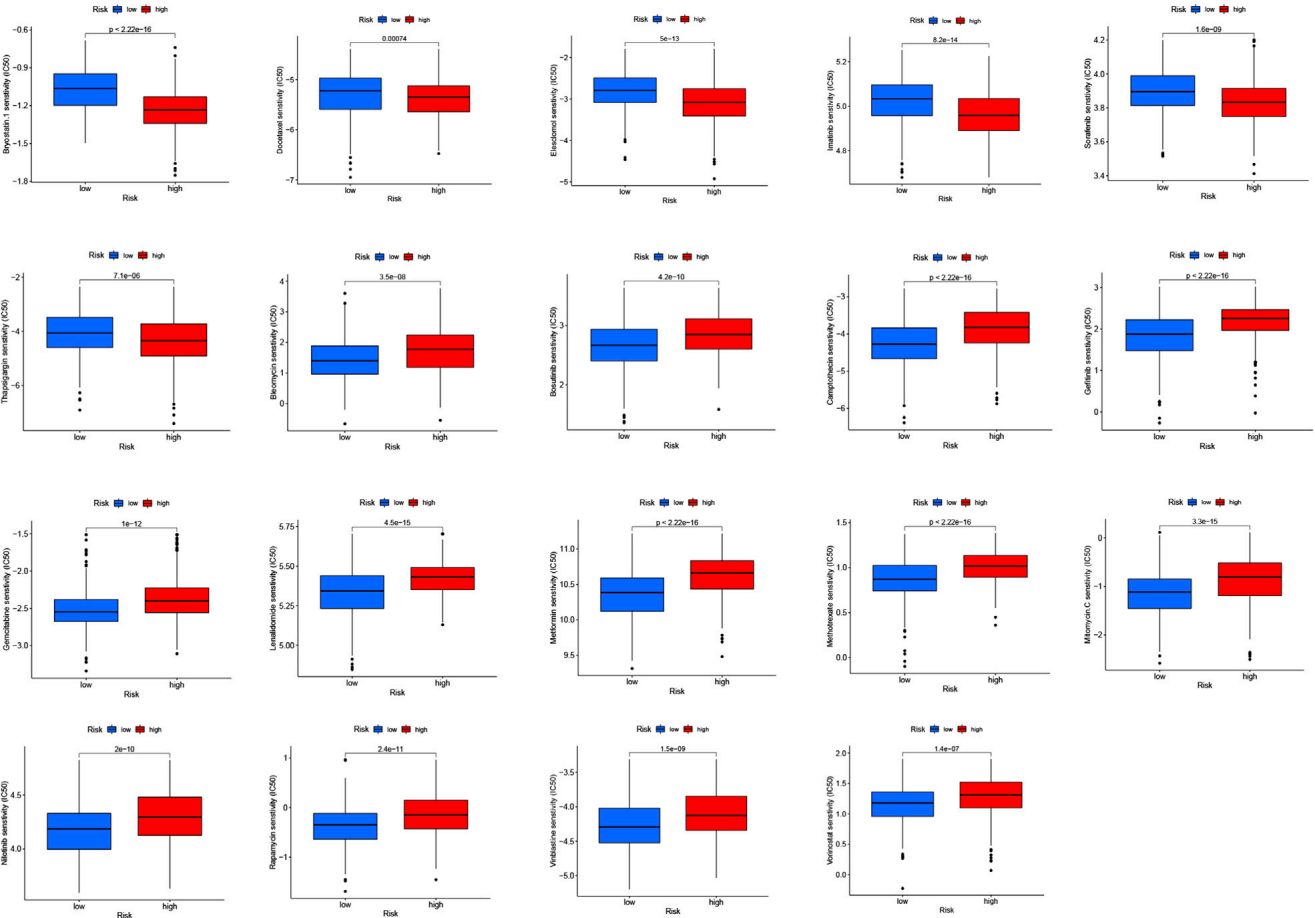
Drug susceptibility (IC50) was associated with two risk groups for melanoma.

To explore the possible application of cuproptosis-related differential genes in individualized therapy of SKCM, we explored the connection between IC50 and risk score of drugs in SKCM therapy. Drug sensitivity analysis showed that bryostatin.1, docetaxel, elesclomol, imatinib, sorafenib and thapsigargin were more sensitive in the high-risk group. However, bleomycin, bosutinib, camptothecin, gefitinib, gemcitabine, lenalidomide, metformin, methotrexate, mitomycin. C, nilotinib, rapamycin, vinblastine and vorinostat were more sensitive in the low-risk group (Figure 10).

### Verification of expression of the seven cuproptosis-related differential genes

**FIGURE 11:**
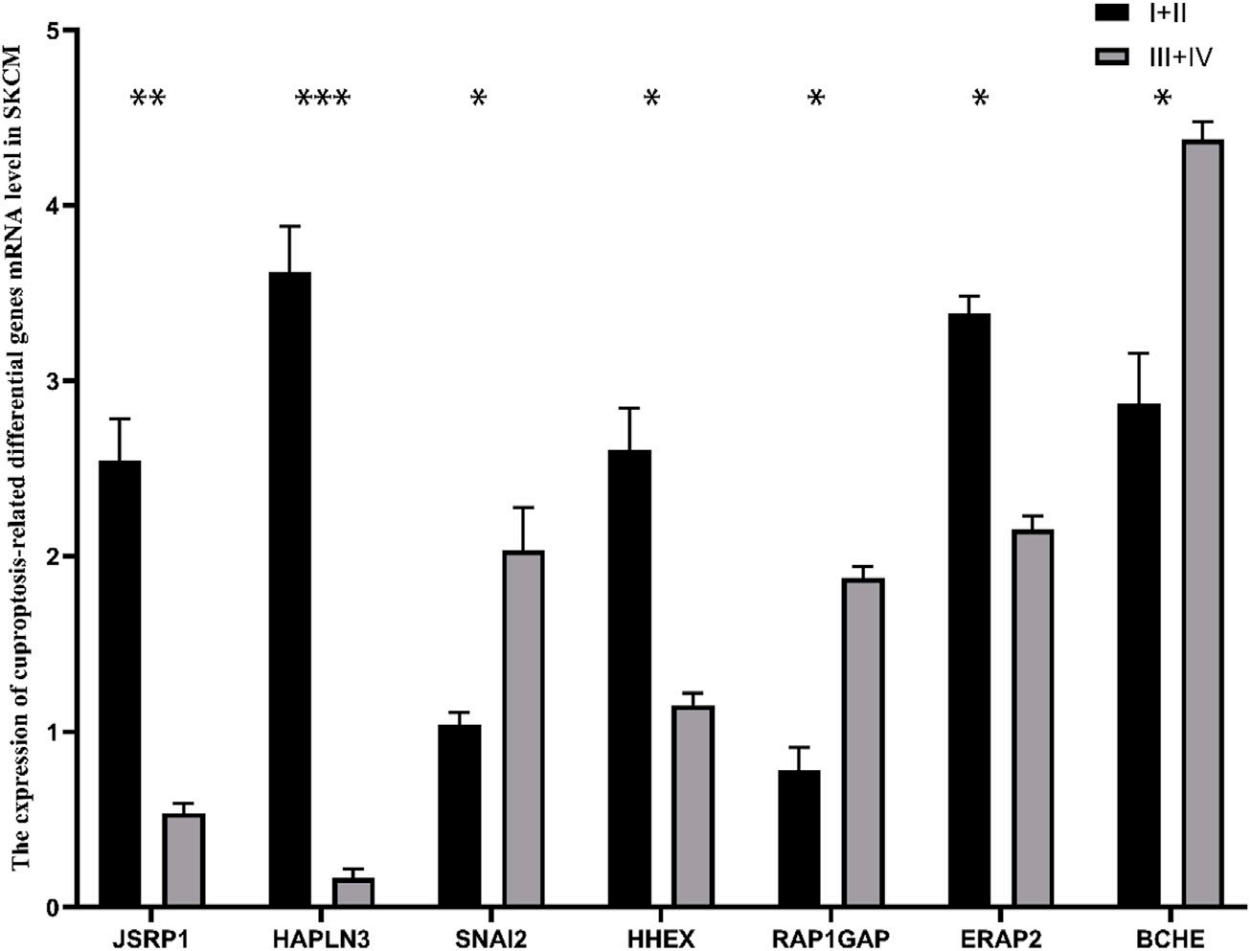
The mRNA expression of 7 cuproptosis-related differential genes in patients with different stages of cutaneous melanoma. *p < 0.05, **p < 0.01, ***p < 0.001.

To ulteriorly Validate the expression levels of the seven cuproptosis-related differential genes, we collected specimens from 11 patients with stage Ⅰ+Ⅱ melanoma and 9 patients with stage Ⅲ+Ⅳ melanoma. The mRNA expression level of SNAI2, RAP1GAP and BCHE in stage Ⅰ+Ⅱ SKCM was strikingly lower than that in stage Ⅲ+Ⅳ SKCM (*p <* 0.05), while the expression of JSRP1, HAPLN3, HHEX and ERAP2 in stage Ⅰ+Ⅱ SKCM patients was significantly higher than stablished (*p <* 0.05) (Figure 11).

## Discussion

SKCM is one of the deadliest malignancies and prone to metastasis. Although chemotherapy, immunotherapy and molecular therapy are available, the prognosis for SKCM patients remains poor, with a very short median survival time (Donizy et al., 2022; Voglis et al., 2022; Zhang et al., 2022). Although there are a variety of clinical tools to predict the prognosis of patients with SKCM (Weiss et al., 2015), in view of the clinical and biological heterogeneity of primary SKCM, new methods or models that more accurately predict the prognosis of patients with SKCM are still needed. To promote the programmed death of melanoma cells has been the direction of clinicians’ efforts. Promoting ferroptosis in melanoma can enhance the sensitivity of melanoma to PD-1 (Guo et al., 2022). Ferroptosis is defined as an iron-dependent and unrestricted form of cell death featured with lipid peroxidation (Dixon Scott et al., 2012). Startlingly, Tsvetkov et al. (2022) recently proposed that copper induces cell death by targeting lapidated TCA cycling proteins, called cuproptosis, which as a novel regulated cell death distinct from apoptosis, necroptosis and ferroptosis (Tang et al., 2022). We conclude that cuproptosis-related genes play a crucial part in the development and prognosis of SKCM.

In our study, we detailedly analyzed the expression of cuproptosis-related differential genes in SKCM and verified them in different stages of SKCM. Their predictive significance in SKCM and their correlation with tumor mutation load and immune function were also analyzed. Firstly, the RNA transcription group data of 469 patients with SKCM was extracted from the TCGA database, and 17 cuproptosis-related genes were selected according to survival analysis of 19 cuproptosis-related genes. Next, RNA transcriptome data for 683 SKCM patients were obtained from GEO and TCGA databases, and 767 cuproptosis-related differential genes related to prognosis of cuproptosis were identified. GO analysis showed that T cell activity, immune receptor activity and external side of plasma membrane were enhanced significantly. Through KEGG pathway analysis, we found that T cell receptor signaling pathway, chemokine signaling pathway, cytokine-cytokine receptor interaction, natural killer cell-mediated cytotoxicity, T cell receptor signaling pathway, B cell receptor signaling pathway and other functions were significantly enhanced. T cell activation is a critical incident both in antiviral and antitumor adaptive immunity (Wang et al., 2022). However, malignant melanoma belongs to the most immunogenic tumor, which can evade T cell recognition by down-regulating tumor associated antigens, defects in antigen processing mechanism, and downregulation of MHC molecules caused by β2-microglobulin mutation, leading to immune evasion (Marzagalli et al., 2019). Moreover, melanoma cells secrete cytokines and chemokines through overactivation of the NF-κB signaling pathway to impede the T-cell targeting of tumor cells (Ellis and Hicklin, 2008; Umansky and Sevko, 2012). NK cells are an important part of initial immunity, which can remove senescent cells and pathogenic microorganisms (Mehta et al., 2018). NK cells are not only more cytotoxic to tumor cells that downregulate MHC expression to evade acquired immunity, but also can directly exert antitumor effects by mobilizing dendritic cells and macrophages and other immune cells or secreting cytokines (Myers and Miller, 2021). At present, new targets and candidate drugs for SKCM immunotherapy are emerging constantly, but most of them are in the early stage, and some of them have unsatisfactory efficacy as monotherapy (Wang et al., 2022). Cuproptosis may play an crucial role in the metastasis and immune escape of melanoma, which could be a new potential target for the therapy of SKCM in the future.

Next, Lasso regression and multi-variable COX regression of 767 cuproptosis-related differential genes were screened out of 7 different differential genes related to cuproptosis. The connection between 7 differential genes and cuproptosis-related genes was verified by relevant analysis. SNAI2 has been implicated in the diseases of melanocytes development and a variety of cancers (Cobaleda et al., 2007). SNAI2 plays a crucial role in regulating T-cell lineages (Pioli et al., 2016) and cancer cell stemness (Chen et al., 2021; Peng et al., 2022), as well as modulating lapatinib resistance in HER2-positive breast cancer (Hamalian et al., 2021) and enhancing 5-fluorouracil sensitivity in colorectal cancer (Findlay et al., 2014). We developed a risk-scoring model for SKCM patients according to seven cuproptosis-related differential genes, using a training set to distinguish between low -risk and high -risk groups. The survival rate of patients in the low-risk group was significantly higher than that in the high-risk group, which was consistent among the training group, test group and all samples group. Importantly, in our risk scoring model, 0.669, 0.669 and 0.685 are ROC values of 1 year, 3 years and 5 years respectively. Indicating that the scoring model can accurately predict the long-term survival outcome of melanoma patients. In addition, we also studied the correlation for the two groups with immune cells, and found that T cells and macrophages were mainly related. This is also consistent with our functional enrichment analysis. Finally, according to the drug sensitivity analysis, we screened 19 drugs with different sensitivities in the two risk groups, which may provide guidance for the treatment of melanoma. Song et al. (2022a), Song et al. (2022b) utilized coagulation and apoptosis related genes to better predict the prognosis and tumor microenvironment of cutaneous melanoma. Although the recent researches on the model of cuproptosis predicting the prognosis of melanoma has shown a good prediction effect, it has not been verified *in vivo* or *in vitro*, and the true prediction effect of the model is still uncertain (Liu et al., 2022; Zhou et al., 2022). We further demonstrated the accuracy and reliability of our model by verifying the expression of seven cuproptosis-related differential genes in different stages cutaneous melanoma patients.

Cuproptosis is a new type of cell death, which may be a breakthrough point for SKCM treatment. However, the research has some limitations. First, the mechanism of cuproptosis in SKCM has not been resolved. In addition, the accurate mechanism between cuproptosis and immune cell infiltration is unclear. Second, the data of our prognosis model comes from the public database, and the lack of additional *in vivo* validation data. Therefore, further basic research and clinical research need to be explored.

## Conclusion

In conclusion, we suggest that cuproptosis can not only regulate the tumor immune microenvironment but also affect the prognosis of SKCM patients, and may offer a basic theory for SKCM patients survival studies and clinical decision-making with potentially therapeutic drugs.

## Data Availability

The datasets presented in this study can be found in online repositories. The names of the repository/repositories and accession number(s) can be found in the article/Supplementary Material.
