# Supplementary figures and images for: The value of cuproptosis-related differential genes in guiding prognosis and immune status in patients with skin cutaneous melanoma
Source: Front Pharmacol. 2023 Apr 17;14:1129544. doi: 10.3389/fphar.2023.1129544 (PMC10149708; doi:10.3389/fphar.2023.1129544)

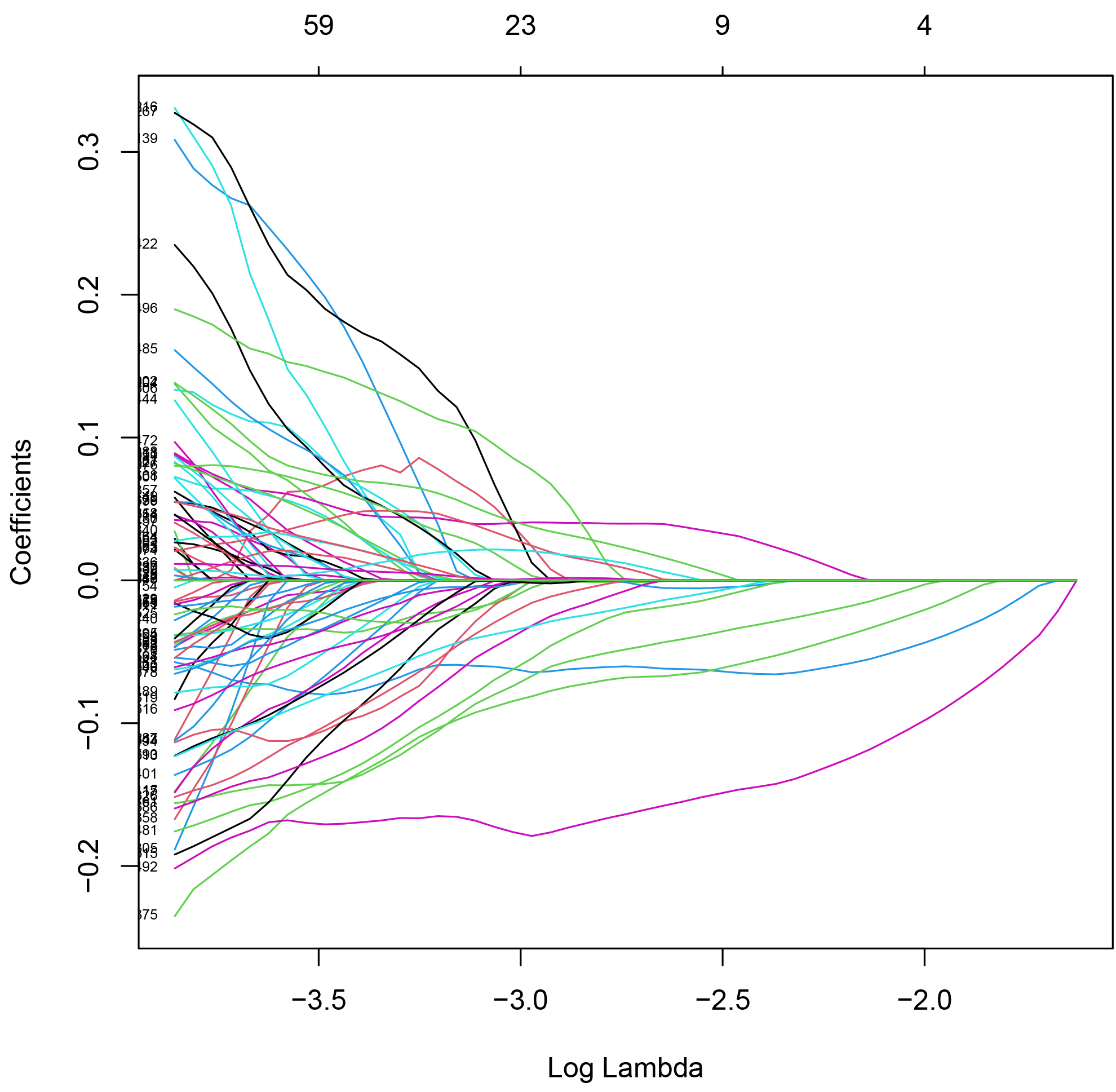

Supplement: Supplementary file 1 [file Image3.jpg]

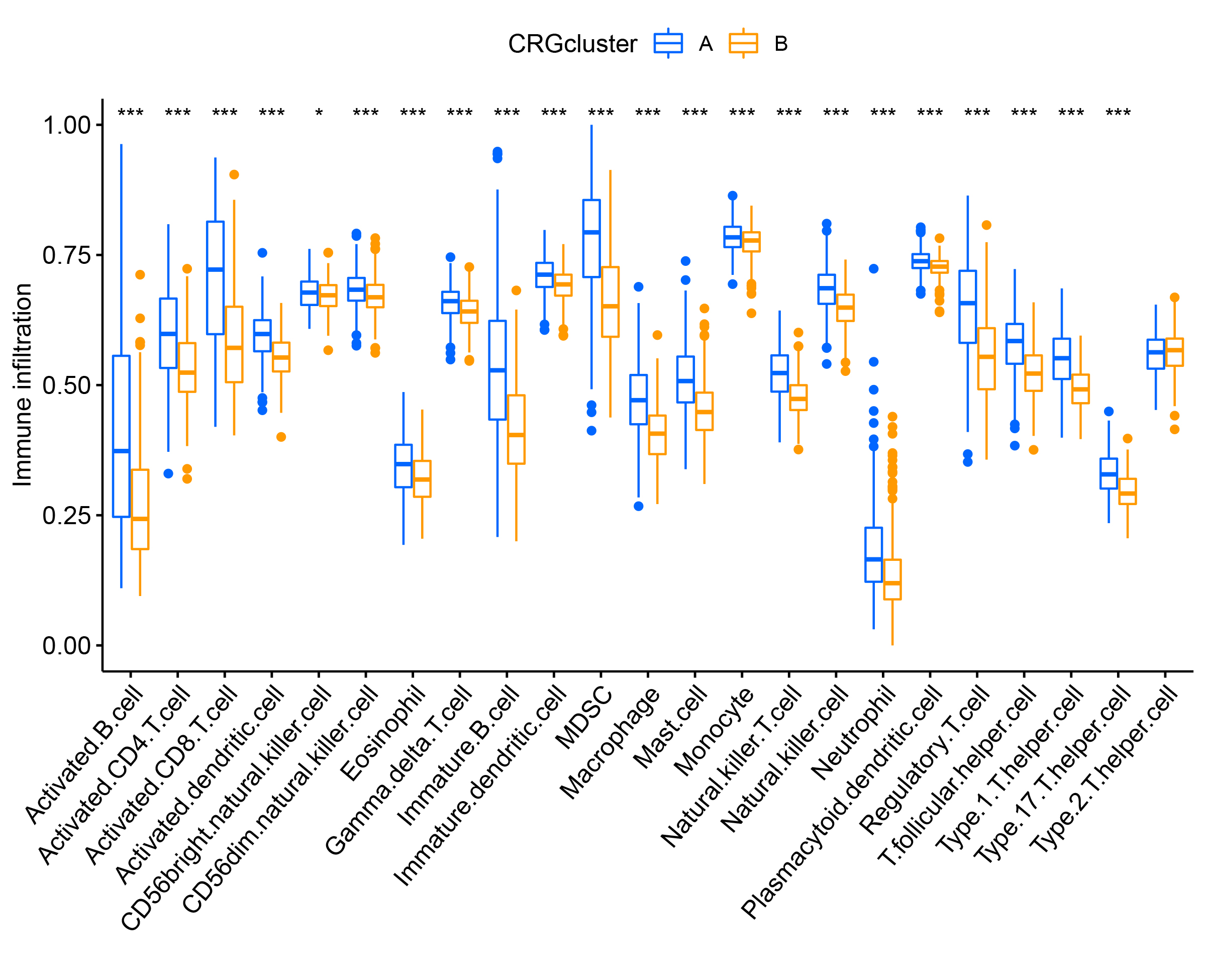

Supplement: Supplementary file 2 [file Image2.jpg]

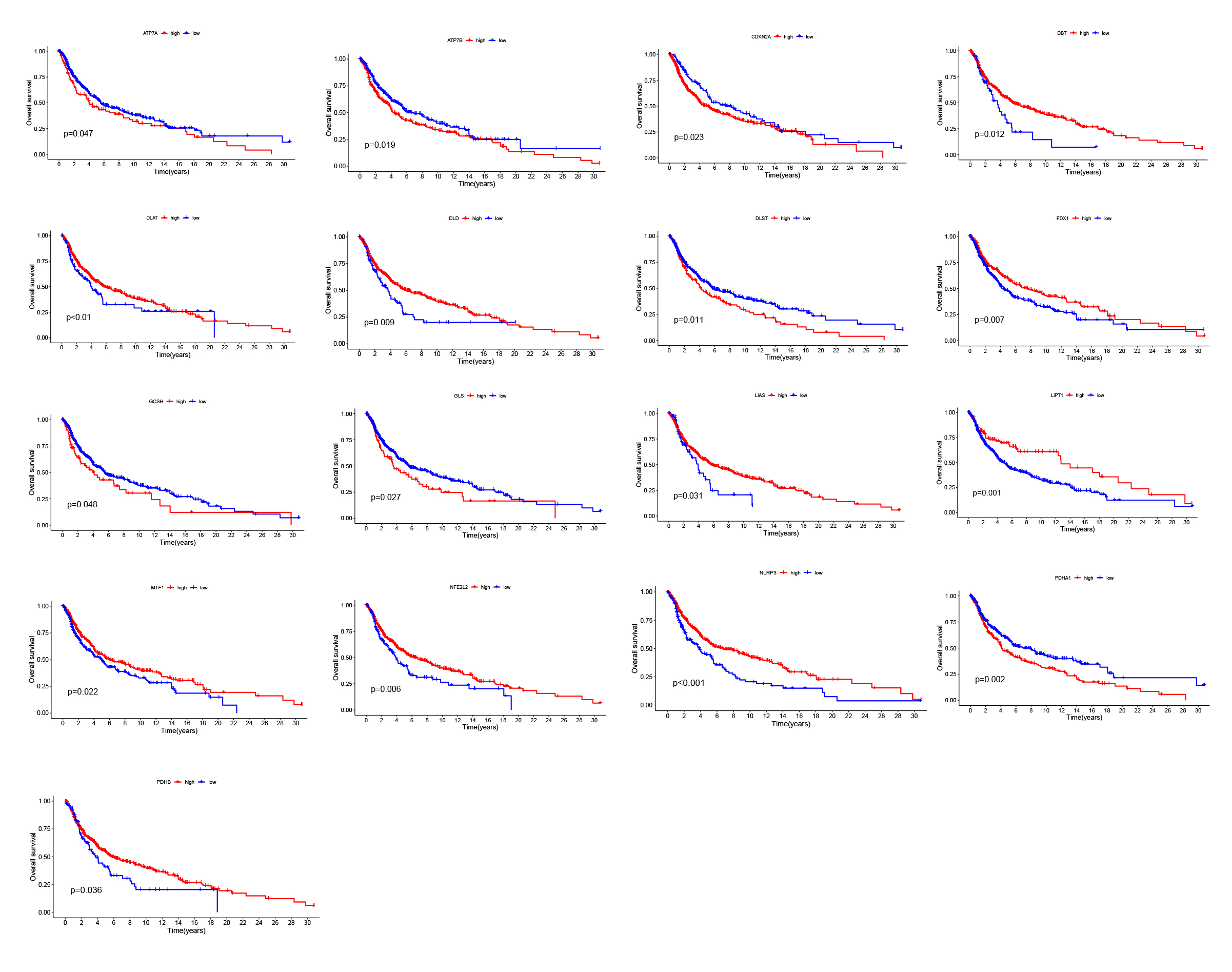

Supplement: Supplementary file 3 [file Image1.jpg]
